# Abundance and dispersal of *Heteronychus arator* (Coleoptera: Scarabaeidae) in maize fields under different fertilizer treatments

**DOI:** 10.1186/s40064-016-1847-8

**Published:** 2016-02-25

**Authors:** M. Abdallah, M. W. Mwatawala, A. B. Kudra

**Affiliations:** Department of Crop Science and Production, Sokoine University of Agriculture, P.O. Box 3005, Morogoro, Tanzania

**Keywords:** *Heteronychus arator*, Spatial, Temporal, Fertilizer

## Abstract

The invasive African black beetle *Heteronychus arator* F. is a serious pest of crops in Tropical and sub Tropical regions, including East Africa. Literature on ecology of this pest in Sub Saharan Africa is scarce. Dispersal and abundance of *H. arator* were determined in maize fields in Njombe Region, in the Southern highlands of Tanzania, from December 2013 to May 2014. Adults of *H. arator* were collected monthly by hand picking and pitfall traps. Results showed that larvae were present throughout the growing season, with low population at planting and peak period coinciding with optimum soil moisture. The abundance of adults varied with time, with high peaks recorded in January. Abundance of both larvae and adults were significantly affected by fertilizer type, with high populations in farmyard manure. The type of fertilizer significantly affected dispersal of *H. arator*. A high number of beetles moved significantly longer distances in farmyard and inorganic fertilizer compared to bioslurry a day after release. Abundance of of beetles was negatively correlated with rainfall but positively correlated with average temperature, while abundance of larvae was positively correlated with rainfall. The results provide useful input into management of *H. arator* under different fertilizer practices and weather conditions.

## Background

The African black beetle *Heteronychus arator* F. is a polyphagous insect attacking a wide range of cultivated crops, with a high preference for pastures (King et al. 1981; Matthiessen et al. 1997). Preferred hosts include potato, (*Solanum tuberosum* L.), Maize (Zea *mays* L.), pineapple (*Ananas comosus* (L.) Merr.), and vegetables such as pea (*Pisum sativum* L.) and tomato (*Lycopersicon esculentum* Mill) (Venter and Louw 1978; Smith et al. 1995; Sinclair et al. 1997; Toit et al. 1997; Matthiessen and Learmonth 1998). *Heteronychus arator* originated from South Africa (Venter and Louw 1978), and invaded South America and the Oceania. In Africa, *H. arator* has been recorded in Eastern and Southern countries, including Namibia, Zambia, Malawi, Mozambique, Botswana, Democratic Republic of Congo and Tanzania.

*Heteronychus arator* attacks various crops during various stages of growth, from seedling to maturity (Ahad and Bhagat 2012). All life stages of *H. arator* are subterranean but adults can fly (King et al. 1981). Young larvae feed on soil organic matter, while more mature larvae attack plant roots. Losses of up to 20–30 % of sown maize (Drinkwater 1987) and up to 70 % of potato (Venter and Louw 1978; Matthiessen and Learmonth 1995) have been reported. Each adult *H. arator* can attack 2–5 tubers of potato (Matthiessen and Learmonth 1995). It is a pest of phytosanitary significance and it has caused great losses where it has been introduced.

Abundance, dispersal, distribution and damage by *H. arator* are dependent on various ecological factors, including soil temperature, moisture and diseases (King et al. 1981). Temperatures above 15 °C are most favourable for development and survival of *H. arator,* with optimum larval development occurring at 20–25 °C (King et al. 1981). Low temperatures limit larval survival (King and Watson 1982).

Phytophagous insects like *H. arator* are subject to many selective pressures, including the abundance and diversity of plants and their spatial and temporal fluctuations. Knowledge on dispersal and abundance of *H. arator* is limited to the Oceania and South Africa. Quantifying dispersal is key to understanding population dynamics of insects and tracking changes in environmental conditions (Roderick and Caldwell 1992).

Insects’ dispersal may be caused by deterioration of habitat, resource depletion, competition or combination of these (Dingle 2001). Likewise, dispersal may be triggered by food availability, population density or environmental factors such as temperature, humidity and rainfall (Danthanarayana 1986). Crops provide a temporal and spatial concentration of resources (Lombaert et al. 2006), which can attract or arrest large number of insects from long distances. A homogeneous crop would provide a high-level of resources to consumers compared to mixed, patchy cropping (Lombaert et al. 2006).

*Heteronychus arator* is an emerging serious pest of maize in Njombe Region, Tanzania. The pest has not been studied in Tanzania, although farmers have reported high damages. Various control methods have been tested (Ball et al. 1997; Erasmus and Van den Berg 2014; Potter et al. 1996; Koppenhöfer et al. 2000).

Farmers who use farmyard manure and bio slurry in maize fields reported high losses due to *H. arator* in Southern highlands of Tanzania. In this study, it was hypothesized that use of manure and bio slurry increased abundance and dispersal of *H. arator* in maize fields. However, this was not determined by research. To understand factors affecting dispersal and abundance of *H. arator* is important before formulating a sound management program. We studied dispersal spatial and temporal abundance of the beetle in patchy maize fields in Southern highlands of Tanzania in relation to fertilizer application.

## Methods

### Description of the study area

Experiments were conducted in Njombe and Wanging’ombe Districts, Njombe Region in the Southern highlands of Tanzania. Njombe is one of the major maize producing regions of Tanzania. We selected four locations, one in Wanging’ombe and three in Njombe district, based on high incidence of *H. arator*. All the locations are characterized by a uni-modal rainfall pattern, with the rainy season starting from November/December to April/May. The area receives an average annual rainfall of 1500 mm, while the average temperature is16 °C per annum. The locations are described in Table 1.

**Table 1:** Description of study locations

District	Village	Location	Altitude (m a.s.l)
Wanging’ombe	Nyumbanitu	S09°14.970′; E034°40.822′	1994
Njombe	Nyombo	S09°3.038′; E034°48.845′	1809
	Mtwango	S09°02.697′; E034°48.648′	1800
	Ibumila	S09° 11.595^′;^ E034°8.513^′^	1840

### Larval abundance

The experiment was set in a Randomised Complete Block Design (RCBD) in four locations. The three treatments viz., inorganic fertilizer, bio slurry and farm yard manure (Table 2) were applied in individual plots replicated in three locations. The size of the plot for each treatment was 10 × 5 m (50 m^2^) with 10 m strips between plots and blocks. The land was prepared in late November and sowing of maize variety PAN 691 (PANNAR Seed Co. Ltd) was done in early December 2013. We established 266 plants/50 m^2^, at a spacing of 75 cm × 25 cm giving a population of 53,200 plants/ha. All standard agronomic practices were followed in each location.

**Table 2:** Fertilizer treatments used in the trial

Treatment	Fertilizer type	Content	Application rate	Timing of application
Inorganic fertilizer	Yara Mila cereal	23 % N, 10 % P_2_O_5_, 5 % K_2_O, 5 % MgO, 3 % S, and 0.3 % Zn	526 kg YMC/ha	At planting
	Yara Vera	46 % N	40 kg N/ha	4 weeks after planting
	Yara Mila Java	23 % N, 6 % S	74 kg YMJ/ha	12 weeks After planting
Bio slurry	Bio slurry manure	1.8 % N, 1.5 % P_2_O_5_, 1.7 % K_2_O	8000 kg/ha	At planting
	Bio slurry leachate	1.8 % N, 1.5 % P_2_O_5_, 1 % K_2_O	10,000 L/ha	4 weeks after planting
FYM	Farmyard manure	0.8 % N,0.5 % P_2_O_5_, 0.7 % K_2_O	10,000 kg/ha	At planting

Larval abundance was determined from a soil sample extracted around the roots (20 × 20 × 20 cm) of three selected plants in a plot (Ahad and Bhagat 2012). Sampling was done monthly, from December 2013 to May 2014. The larvae in each soil sample were counted and recorded. We recorded mean number of larvae per plot per month.

### Adult abundance and dispersal

Beetles used in this study were collected by hand picking and by trapping every sampling month from January to May. Collection of insects was done in the morning and release was done at night between 700 h to 900 h (Nyundo and Yarro 2007).We used plastic pitfall traps (15 × 13 × 8 cm; length, top diameter, bottom diameter), half filled with partially decomposed farmyard manure. Collected insects were kept in semi-transparent plastic boxes (30 cm × 30.5 cm × 25 cm), filled with farmyard manure, germinated maize and beans seedlings as food. The elytra of beetles were marked by triangular notching on posterior end one day before the release as suggested by Guzman et al. (2011).

### Abundance estimation

A capture—mark-recapture (CMR) study was conducted during the 2013–2014 cropping season, in uniformly established maize plots within Ibumila village (Table 1). Three plots of 40 m × 45 m were treated with fertilizers as described in Table 2 and replicated in three locations. Distance between plots was 50 m. A total of 3400 marked beetles were released at each instance for abundance estimation as described by Arakaki et al. (2008). Beetles were released in the night (Nyundo and Yarro 2007) once per month and were recaptured for three consecutive days after each release. Adults were recaptured by hand picking and trapping. Numbers of recaptured beetles were pooled across distance for each plot, each month. We recorded, for each plot, number of released (marked) beetles, number of recaptured beetles and number of unmarked beetles.

Lincoln index was used to determine absolute abundance which is the variation of Jolly-Seber (Bancroft 2005).

$${\text{N}} = {\text{ M }}\left( {{\text{U}} + 1} \right)/{\text{R}} + 1$$

where N is the population estimate, M is the number of marked beetles, U is the number of unmarked beetles and R is the number of recapture bugs that were marked. The population estimates depends on the ratio of marked to unmarked beetles that removes bias that might arise due to capture efficiency. To balance the sampling efforts each plot was measured by the total time taken. The effects of distance were corrected by pooling recaptures across distance for each month.

### Dispersal estimation

A total of 1800 marked beetles were released once every month, for dispersal estimation. Release and recapture were done from January to May 2014, as described above. Beetles were recaptured at 2, 4, 16, and 32 m radii from the release point. We recorded and compared the number of beetles caught at each radius from the release point.

### Data analysis

General Linear Model was used to analyse abundance and dispersal of beetles using R statistical package. Two-way analysis of variance (ANOVA) was used to analyse abundance of larvae and beetles, with fertilizer and month as factors. Dispersal was analysed by three factors ANOVA, with as fertilizer type, days after release as the sub factor and distance from release point as factors. Post Hoc Tukey Test was used to compare means. Correlation coefficients for adult and larvae counts with selected weather parameters were calculated using Pearson correlation method under SPSS version 16 and evaluated for significance as suggested by Ahad and Bhagat (2012).

## Results and discussion

### Results

**Fig. 1:**
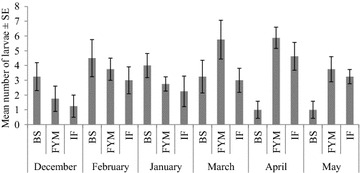
Mean number of of larvae of *H. arator* in plots treated with different types of fertilizers

Temporal variation in larval abundance in maize plots under different fertilizer treatment is presented in Fig. 1. Two way ANOVA results showed that larval abundance was not significantly affected by fertilizer type (F_(2, 54)_ = 2.93, p < 0.06) or month F_(5, 54)_ = 2.29, p < 0.058). However, the interaction between fertilizer type and month significantly affected abundance of larvae F_(10, 554)_ = 2.88, p < 0.005). Analysis of simple effects of fertilizers did not show significant variations in larval abundance in December (F_(2, 9)_ = 1.48, p = 0.28), January (F_(2, 9)_ = 1.24, p = 0.33), February F_(2, 9)_ = 0.57, p = 0.58) or March F_(2, 9)_ = 1.91, p = 0.2). Significant simple effects of fertilizers were observed in April F_(2, 9)_ = 10.22, p = 0.004) and May F_(2, 9)_ = 4.98, p = 0.035).

Multiple comparison of means (Tukey, 95 % CI) showed significant differences between bioslurry and farmyard manure (p = 0.005), as well as bioslurry and inorganic fertilizer (p = 0.02) in April. Farmyard manure and bioslurry were not significantly different (p = 0.597). Bioslurry also differed significantly with farmyard manure in May (p = 0.04) but not with inorganic fertilizer (p = 0.088). Farmyard manure and inorganic fertilizer were not significantly different (p = 0.85) in May (See also Fig. 1). Larvae were highly abundant in farmyard manure treated plots, peaking in March and April (Fig. 1).

**Fig. 2:**
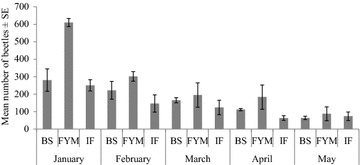
Mean number of of adults of *H. arator* in plots treated with different types of fertilizers (*BS* bioslurry, *FYM* farmyard manure, *IF* inorganic fertilizer) (*BS* Bioslurry, *FYM* farmyard manure, *IF* inorganic fertilizer)

Figure 2 presents temporal variation in beetles’ abundance in maize plots under different fertilizer treatments. The number of collected adults of *H. arator* varied significantly among plots, depending on type of fertilizer (F_(2, 30)_ = 9.31, p < 0.001), and month (F_(4, 30)_ = 14.12, p < 0.001). The interaction between fertilizer type and month did not significantly affect beetles’ abundance F_(8, 30)_ = 1.9, p = 0.09) (see also Fig. 2). Significant main effects on beetles’ abundance were observed among fertilizers (F_(2, 6)_ = 5.66, p = 0.04) and month (F_(4, 10)_ = 10.35, p = 0.001). Highest marginal mean (fertilizer effects averaged over months) was recorded in farmyard manure (274 ± 20), followed by bioslurry (196 ± 22) and inorganic fertilizer (130 ± 27). Post Hoc Tukey test (95 % CI) on marginal means revealed significant differences (p = 0.04) in beetles abundance between inorganic fertilizer and farmyard manure, but not between inorganic fertilizer and bioslurry (p = 0.695) or bioslurry and farmyard manure (p = 0.11).

**Fig. 3:**
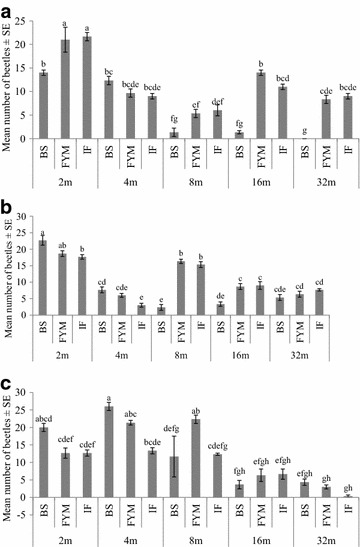
Mean number of beetles caught at various distances from release point in plots under different fertilizer treatments **a** a day after release **b** 2 days after release **c** 3 days after release. (*BS* bioslurry, *FYM* farmyard manure, *IF* inorganic fertilizer). Mean with the *same letters* are not significantly different (Tukey test, 95 % CI)

Distance covered by beetles increased with time since release (Fig. 3). Three factors ANOVA results showed that number of recaptured beetles differed significantly with fertilizer type (F_(2, 90)_ = 19.22, p < 0.001), distance from point of release (F_(4, 90)_ = 92.82, p < 0.001) and days since release (F_(2, 90)_ = 11.92, p < 0.001). The interaction between fertilizer, days since release and distance from release point was also significant (F_(16, 90)_ = 11.92, p < 0.001). Analysis further showed significant simple interactions between fertilizer and distance from release point, after day 1 F_(2, 30)_ = 32.5), day 2 F_(2, 30)_ = 24.98, p = 0.001), and day 3 (F_(2, 30)_ = 5.51, p = 0.001) since release. Post hoc Tukey test (95 % CIs) showed that significantly less numbers of beetles were caught in bioslurry plots at longer distances from release point day after release. Significantly more beetles were caught within the release point in farmyard manure and inorganic fertilizer plots, a day after release (Fig. 3).

Poisson regression results showed significant associations between rainfall, average temperature and adults abundance (Table 3). The number of beetles significantly decreased with increase in rainfall (e = −0.011, p < 0.001, Exp [β] = 0.989). On the contrary, average temperature was positively associated with number of beetles [e = 0.748, p < 0.001, Exp (β) = 2.11]. Relative humidity had no significant effect on beetles’ abundance (Table 3). Larval abundance was significantly associated with rainfall [e = 0.0045, p < 0.03, Exp (β) = 1.004], but not with relative humidity and average temperature (Table 4).

**Table 3:** Correlation between number of adults and weather parameters

Parameter	e	SE	t (*)	Tpr	Exp (β)
RH	0.367	0.204	1.80	0.072	1.444
Rainfall	−0.01112	0.00218	−5.10	<.001	0.9889
Av temperature	0.748	0.166	4.50	<.001	2.112

*e* coefficient of parameters, *SE* standard error of estimates, t (*) standard error, *tpr* probability, *Exp (β)* exponent of estimate

**Table 4:** Correlation between number of larvae and weather parameters

Parameter	e	SE	t (*)	Tpr	Exp (β)
RH	0.577	0.368	1.57	0.117	1.780
Rainfall	0.00449	0.00207	2.17	0.030	1.004
Av temperature	0.029	0.100	0.29	0.773	1.029

*e* coefficient of parameters, *SE* standard error of estimates, t (*) standard error, *tpr* probability, *Exp (β)* exponent of estimate

## Discussion

This study presents the abundance and dispersal of the invasive *H. arator* in soils under different fertilizer management. Our results revealed dependence of larval and beetles’ abundance on fertilizer type. We also observed temporal variations in larval and beetles abundance, with high numbers in farmyard manure treated plots. Sara et al. (2013) suggested that spatial activities of beetles are greatly influenced by change in habitat. Adults and white grubs of green jute beetle were reported to be attracted to the field fertilized with farmyard manure from cow and poultry (Diagne 2004).

Dispersal of *H. arator* from the release point increased with time. Schumann and Vidal (2012) reported that dispersal of the Western Corn Borer larvae increased as they developed and the larvae moved off their original place of emergence and into deeper soil layers. Ross and Ostlie (1990) reported mean dispersal distance *Ostrinia nubilalis* (Hübner) increased linearly with time. Glogoza et al. (1998) observed a positive association between the probability of finding *Phyllophaga implicita* (Horn) (Coleoptera: Scarabaeidae) and the distance from the food source.

Results from the current study showed that temperature, and rainfall played a major role on temporal abundance of *H. arator.* Ahad and Bhagat (2012) reported that the population of larvae (white grubs) is determined by rainfall and soil moisture. East et al. (1981) reported that black maize beetle was a persistent problem during favourable environmental conditions such as rainfall, temperature, relative humidity and good soil types. Soil moisture also plays a significant role in burrowing depth of *H. arator*, with deeper burrows associated with high level of soil moisture. Further, both organic matter and soil moisture were reported to influence the selection of habitat for burrowing and oviposition by many beetles (Diagne 2004).

The abundance of adults was positively correlated with temperature, but negatively, correlated with rainfall. On other hand, the abundance for larvae was positively correlated with rainfall but not temperature and relative humidity. Temperature may be a key driver for spatial and temporal abundance of arthropods (Kearns and Stevenson 2012). Survival and growth rate of *H. arator* are favoured by high temperature and rainfall, as most physiological responses could be triggered by the change in temperature (King and Watson 1982). The influence of temperature on population dynamics of *H.arator* might be modified by other factors such as soil types, level of soil moisture, crop composition and management practices (East et al. 1981).

According to East et al. (1981) *H. arator* adults can undergo extensive dispersal flights due to high mobility imposed for oviposition during dry season. Diagne (2004) pointed out that during the periods of mating the adults tend to avoid hard soils with little organic matter content and concentrate in areas with soft soils which are rich in organic matter, and this biological behaviour has influence on the extent of beetles dispersal. Early flights appear to be initiated by the first rainfall, as adults emerge from pupae (Bell et al. 2011).

## Conclusion

This study provided the basic information for *H. arator* on maize, in trials done in one season cycle. Abundance of black maize beetle is dependent on type of fertilizer as well as climatic factors, notably rainfall, relative humidity and temperature. Timely application of fertilizer is necessary to ensure growth of strong plants that can withstand attacks by the pest. Future multi seasonal studies are recommended.
